# Crystal structure of an ordered [WOF_5_]^−^ salt: (1,10-phen-H)[WOF_5_] (1,10-phen = 1,10-phenanthroline)

**DOI:** 10.1107/S2056989020009767

**Published:** 2020-07-24

**Authors:** Douglas Turnbull, Michael Gerken

**Affiliations:** a Canadian Centre for Research in Advanced Fluorine Technologies, Department of Chemistry and Biochemistry, University of Lethbridge, 4401 University Drive West, Lethbridge, Alberta, Canada, T1K 3M4

**Keywords:** tungsten, fluoride, oxide, crystal structure, O/F ordered anion

## Abstract

Crystals of 1,10-phenanthrolinium penta­fluorido­oxidotungstate(VI), (1,10-phen-H)[WOF_5_] (1,10-phen = 1,10-phenanthroline, C_12_H_8_N_2_), were obtained upon hydrolysis of WF_6_(1,10-phen) in CH_3_CN at 193 K. The (1,10-phen-H)[WOF_5_] salt contains a rare example of a [WOF_5_]^−^ anion in which the oxygen and fluorine atoms are ordered. This ordering was verified by bond-valence determinations and structural comparisons with [Xe_2_F_11_][WOF_5_] and Lewis acid-base adducts of WOF_4_ with main-group donor ligands. The crystal packing is controlled by N—H⋯F hydrogen bonding that is directed exclusively to the axial F atom as a result of its increased basicity caused by the *trans* influence of the oxido ligand.

## Abstract 2

The crystal of structure of (1,10-phen-H)[WOF_5_], a rare example of a [WOF_5_]^−^ salt in which the oxygen and fluorine atoms are ordered, is reported.

## Chemical context   

The crystal structure of tetra­meric, fluorine-bridged WOF_4_ (Edwards & Jones, 1968 ▸), as well as those of various fluorido-oxidotungstates(VI) of the form [WO_*n*_F_6–*n*_]^*n*−^ (*n* = 1–3), are characterized by extensive disorder between the oxido and fluorido ligands (Voit *et al.*, 2006 ▸). Such disorder complications originally led to the incorrect assumption that WOF_4_ existed as an oxygen-bridged species. This was later disproved by vibrational spectroscopic studies of WOF_4_ that revealed exclusively terminal W=O bonds (Bennett *et al.*, 1972 ▸; Asprey *et al.*, 1972 ▸). In [WO_*n*_F_6–*n*_]^*n*−^ anions, the nature of the O/F disorder can be controlled by the properties of the counter-cations. For example, Na[WO_2_F_4_] is ordered (Vlasse *et al.*, 1982 ▸; Chaminade *et al.*, 1986 ▸), whereas Rb[WO_2_F_4_] (Udovenko & Laptash, 2008*a* ▸) and Cs[WO_2_F_4_] (Srivastava *et al.*, 1992 ▸) are statically disordered, and [NH_4_]_2_[WO_2_F_4_] exhibits simultaneous static and dynamic disorder, both of which are quenched below 201 K to reveal an ordered structure (Udovenko & Laptash, 2008*b* ▸). In addition, the double salt, [HNC_6_H_6_OH]_2_[Cu(NC_5_H_5_)_4_][WO_2_F_4_]_2_, exhibits ordered [WO_2_F_4_]^2−^ anions at 153 K as a result of H⋯F and Cu⋯O secondary-bonding inter­actions (Welk *et al.*, 2001 ▸). An ordered [WO_3_F_3_]^3−^ fragment was identified within Pb_5_W_3_O_9_F_10_ (Abrahams *et al.*, 1987 ▸) and, despite extensive dynamic disorder within [NH_4_]_3_[WO_3_F_3_] (Voit *et al.*, 2006 ▸), the stereochemistry could be resolved from the observed displace­ment of the tungsten atoms from their octa­hedral symmetry centres (Udovenko & Laptash, 2008*a* ▸). In all cases, mutual *cis* arrangements of the oxido ligands are preferred (*i.e*., *cis*-[WO_2_F_4_]^2−^ and *fac*-[WO_3_F_3_]^3−^). A *trans* influence from the oxido ligands results in increased electron density on the effected fluorido ligands, and it is these fluorido ligands that participate in fluorine bridging within multinuclear systems, such as in WOF_4_ and in various [W_2_O_2_F_9_]^−^ salts with different counterions {[H_3_O]^+^(Hoskins *et al.*, 1987 ▸); [WF_4_(2,2′-bipy)_2_]^2+^(Arnaudet *et al.*, 1992 ▸); [Os_3_(CO)_12_H]^+^ (Crossman *et al.*, 1996 ▸); [XeF_5_]^+^(Bortolus *et al.*, 2020 ▸); Li–Cs^+^ (Stene *et al.*, 2020 ▸)}, and in [W_2_O_4_F_6_]^2−^ (Wollert *et al.*, 1991 ▸).
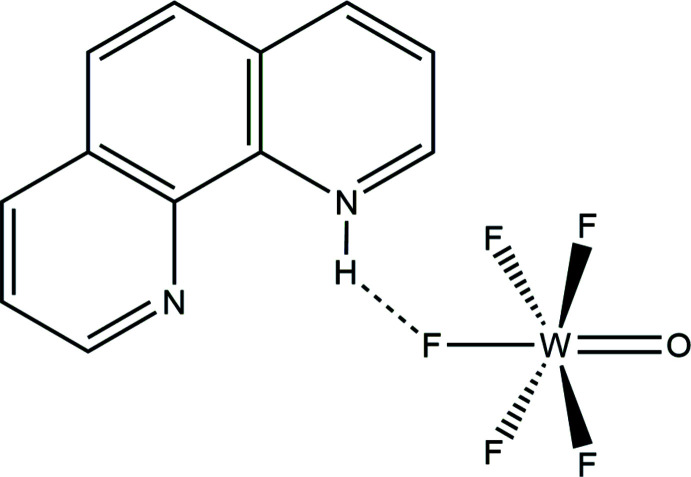


**Figure 1:**
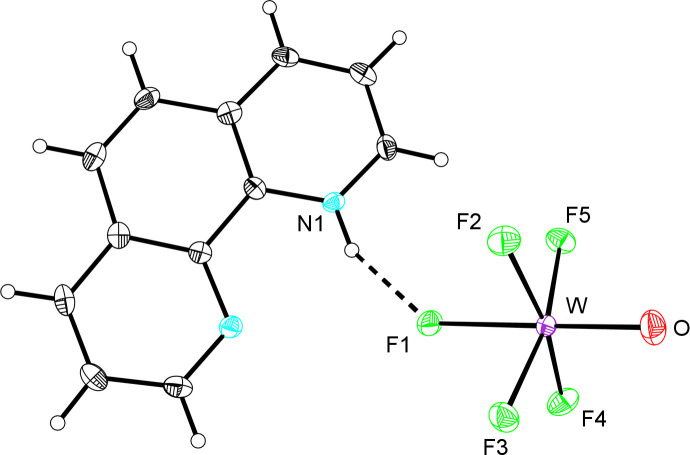
Displacement ellipsoid plot (50% probability level) of (1,10-phen-H)[WOF_5_].

Crystallographically characterized [WOF_5_]^−^ salts with a range of counterions {[As(C_6_H_5_)_4_]^+^ (Massa *et al.*, 1982 ▸); [Cs(15-crown-5)_2_]^+^ (Nuszhär *et al.*, 1992 ▸); Ag^2+^ (Mazej *et al.*, 2017 ▸); [WF_4_(1,2-{P(CH_3_)_2_}_2_C_6_H_4_)]^2+^ (Levason *et al.*, 2018 ▸)} have exhibited some degree of O/F disorder in the anion, obfuscating the W=O and W—F bond lengths. Recently, however, [Xe_2_F_11_][WOF_5_] and [XeF_5_][WOF_5_]·XeOF_4_ were reported to contain [WOF_5_]^−^ anions that are ordered as a consequence of multiple Xe⋯F—W inter­actions (Bortolus *et al.*, 2020 ▸). Herein, we report a new ordered [WOF_5_]^−^ salt in the form of (1,10-phen-H)[WOF_5_] (Fig. 1 ▸), obtained during an attempted crystallization of WF_6_(1,10-phen) (Turnbull *et al.*, 2019*a* ▸).

## Structural commentary   

The W=O bond length in (1,10-phen-H)[WOF_5_] [1.698 (2) Å] is indistinguishable from those in [Xe_2_F_11_][WOF_5_] [1.698 (3) Å; Bortolus *et al.*, 2020 ▸), WOF_4_{OP(C_6_H_5_)_3_} [1.682 (5) Å; Levason *et al.*, 2016 ▸] and WOF_4_(NC_5_H_5_) [1.690 (3) Å; Turnbull *et al.*, 2019*b* ▸]. The W—F_eq_ bond lengths [1.8677 (15)–1.8809 (15) Å] are also insignificantly different from those in WOF_4_{OP(C_6_H_5_)_3_} [1.857 (3)–1.871 (3) Å; Levason *et al.*, 2016 ▸] and WOF_4_(NC_5_H_5_) [1.859 (3)–1.868 (3) Å; Turnbull *et al.*, 2019*b* ▸]. The W—F_eq_ bonds in [Xe_2_F_11_][WOF_5_] (Bortolus *et al.*, 2020 ▸) are also of similar lengths to those in the title compound, although one terminal W—F_eq_ bond [1.848 (2) Å] is significantly shorter in the [Xe_2_F_11_]^+^ salt, and one bridging bond significantly longer [1.900 (2) Å].

The W—F_ax_ bond length of (1,10-phen-H)[WOF_5_] [2.0048 (15) Å] is slightly shorter than those in [Xe_2_F_11_][WOF_5_] [2.047 (2) Å; Bortolus *et al.*, 2020 ▸] and [C_5_H_5_NH][W(NC_6_F_5_)F_5_] [2.0212 (13) Å; Turnbull *et al.*, 2017 ▸]. In the latter compound, the N1—H1⋯F1 inter­action resulted in significant elongation of the W—F_ax_ bond with respect to that observed in [N(CH_3_)_4_][W(NC_6_F_5_)F_5_] [1.973 (3) Å; Turnbull *et al.*, 2017 ▸]. Gas-phase geometry optimizations of the [Xe_2_F_11_][WOF_5_] ion pair and free [WOF_5_]^−^ corroborated a significant elongation of the W—F_ax_ bond in the former anion compared to the latter (2.148 *vs* 1.972 Å, respectively) arising from cation–anion inter­actions (Bortolus *et al.*, 2020 ▸).

The individual bond valences of the [WOF_5_]^−^ anion in (1,10-phen-H)[WOF_5_] (Table 1 ▸) reveal that the W=O bond (*ν* = 1.81) is approximately double the strength of the W—F_eq_ bonds (*ν* = 0.87–0.90), indicating complete O/F ordering; in a disordered anion, the W=O bond valence is artificially decreased due to averaging with the W—F single bonds. The F1 atom possesses a valence sum significantly less than unity (*V* = 0.81) because of the *trans* influence of the oxido ligand and N1—H1⋯F1 hydrogen-bonding inter­action substanti­ally polarizing that bond.

## Supra­molecular features   

**Figure 2:**
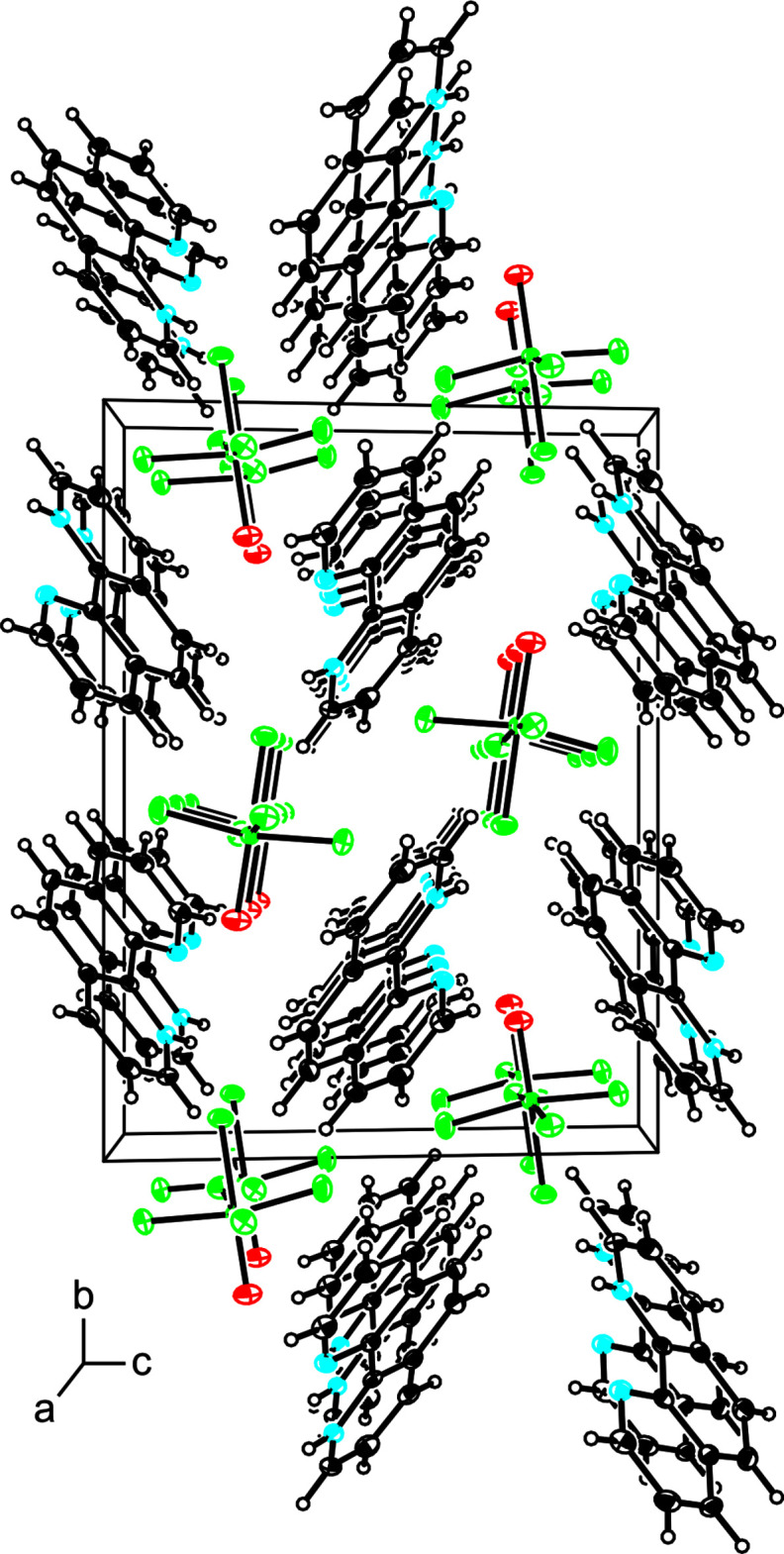
Crystal packing of (1,10-phen-H)[WOF_5_] viewed along the *a* axis.

Besides the N1—H1⋯F1 hydrogen-bonding inter­actions, there also exist weak intermolecular C5⋯O [3.163 (3) Å] and C3⋯C10 [3.204 (4) Å] inter­actions that result in the formation of columns of cations and anions running parallel to the *a* axis (Fig. 2 ▸) in the packed crystal. The crystal packing, however, appears to be dominated by the N1—H1⋯F1 hydrogen bonds (Table 2 ▸) and other inter­molecular inter­actions, such as π–π stacking inter­actions between cations, are absent.

## Synthesis and crystallization   

In the dry box, a 1/4"-o.d. FEP reactor, equipped with a 316-stainless-steel valve and pre-passivated with F_2_ (100%, Linde Gas), was charged with WF_6_(1,10-phen) (*ca* 0.01 g), prepared as described previously (Turnbull *et al.*, 2019*a* ▸). Aceto­nitrile (*ca* 0.1 mL), dried as previously described (Winfield, 1984 ▸), was distilled into the reactor through a glass vacuum line equipped with grease-free PTFE stopcocks (J. Young). The reactor was heated to 353 K in a hot-water bath and allowed to cool to ambient temperature over 16 h. The reactor was then cooled rapidly to 233 K and the solvent was removed under dynamic vacuum at that temperature, resulting in the formation of colourless needles of (1,10-phen-H)[WOF_5_], together with an off-white microcrystalline material that was not further characterized, but is presumed to contain WF_6_(1,10-phen) and (1,10-phen-H)[WOF_5_].

The reactor was cut open and the crystals transferred onto an aluminium trough cooled to 193 K under a constant stream of liquid-N_2_-cooled, dry N_2_. The selected crystal was affixed to a Nylon cryo-loop submerged in perfluorinated polyether oil (Fomblin Z-25) and quickly transferred to the goniometer to minimize exposure to air.

## Refinement details   

Crystallographic data collection and refinement parameters are summarized in Table 3 ▸. All the hydrogen atoms were located in difference Fourier maps and were refined using a riding model, with the exception of H1, the position of which was refined freely (Table 2 ▸).

## Supplementary Material

Crystal structure: contains datablock(s) I. DOI: 10.1107/S2056989020009767/cq2038sup1.cif

Structure factors: contains datablock(s) I. DOI: 10.1107/S2056989020009767/cq2038Isup2.hkl

CCDC reference: 2016898

Additional supporting information:  crystallographic information; 3D view; checkCIF report

## Figures and Tables

**Table 1:** Bond valences and sums of the [WOF_5_]^−^ anion in (1,10-phen-H)[WOF_5_]

	*ν_i_*^*a*^**		*V^*b*^*
		W	5.98
W=O	1.81	O	1.88
W—F1	0.62	F1	0.81
W—F2	0.89	F2	0.95
W—F3	0.88	F3	0.94
W—F4	0.90	F4	0.97
W—F5	0.87	F5	0.95

Notes: (*a*) Defined as *ν_i_* = exp [*R_o_* − *R*/*b*], where *R* is the observed bond length (in Å) and *R_o_* and *b* are empirical parameters (Brown & Altermatt, 1985 ▸; Brese & O’Keeffe, 1991 ▸; Brown, 2002 ▸; Adams *et al.*, 2004 ▸). (*b*) Defined as *V* = Σ(*ν_i_*). Only secondary contacts within the sum of the van der Waals radii (Bondi, 1964 ▸) were considered.

**Table 2:** Hydrogen-bond geometry (Å, °)

*D*—H⋯*A*	*D*—H	H⋯*A*	*D*⋯*A*	*D*—H⋯*A*
N1—H1⋯F1	0.89 (3)	1.88 (3)	2.704 (3)	154 (3)

**Table 3:** Experimental details

Crystal data	
Chemical formula	(C_12_H_9_N_2_)[WOF_5_]
*M*_r_	476.06
Crystal system, space group	Monoclinic, *P*2_1_/*n*
Temperature (K)	112
*a*, *b*, *c* (Å)	7.1664 (2), 15.5088 (4), 11.6516 (4)
β (°)	101.202 (3)
*V* (Å^3^)	1270.31 (7)
*Z*	4
Radiation type	Mo *K*α
μ (mm^−1^)	9.16
Crystal size (mm)	0.27 × 0.12 × 0.06

Data collection	
Diffractometer	Rigaku SuperNova, Dual source (Mo and Cu), Pilatus 200/300K
Absorption correction	Analytical [numerical absorption correction using a multifaceted crystal model based on expressions derived by Clark & Reid (1995 ▸) implemented in *CrysAlis PRO* (Rigaku OD, 2015 ▸). Empirical absorption correction using spherical harmonics, implemented in SCALE3 ABSPACK]
*T*_min_, *T*_max_	0.447, 0.645
No. of measured, independent and observed [*I* > 2σ(*I*)] reflections	15835, 2908, 2652
*R*_int_	0.033
(sin θ/λ)_max_ (Å^−1^)	0.649

Refinement	
*R*[*F* ^2^ > 2σ(*F* ^2^)], *wR*(*F* ^2^), *S*	0.016, 0.038, 1.06
No. of reflections	2908
No. of parameters	194
H-atom treatment	H atoms treated by a mixture of independent and constrained refinement
Δρ_max_, Δρ_min_ (e Å^−3^)	0.97, −0.83

Computer programs: *CrysAlis PRO* (Rigaku OD, 2015 ▸), *SHELXT* (Sheldrick, 2015*a* ▸), *SHELXL* (Sheldrick, 2015*b* ▸), *XP* (Sheldrick, 2008 ▸), *OLEX2* (Dolomanov *et al.*, 2009 ▸) and *publCIF* (Westrip, 2010 ▸).

## supplementary crystallographic information

### Crystal data

**Table:**

(C_12_H_9_N_2_)[WOF_5_]	*F*(000) = 888
*M**_r_* = 476.06	*D*_x_ = 2.489 Mg m^−^^3^
Monoclinic, *P*2_1_/*n*	Mo *K*α radiation, λ = 0.71073 Å
*a* = 7.1664 (2) Å	Cell parameters from 8493 reflections
*b* = 15.5088 (4) Å	θ = 3.6–31.4°
*c* = 11.6516 (4) Å	µ = 9.15 mm^−^^1^
β = 101.202 (3)°	*T* = 112 K
*V* = 1270.31 (7) Å^3^	Needle, clear colourless
*Z* = 4	0.27 × 0.12 × 0.06 mm

### Data collection

**Table:**

Rigaku SuperNova, Dual source (Mo and Cu), Pilatus 200/300K diffractometer	2908 independent reflections
Radiation source: micro-focus sealed X-ray tube, SuperNova (Mo) X-ray Source	2652 reflections with *I* > 2σ(*I*)
Mirror monochromator	*R*_int_ = 0.033
ω scans	θ_max_ = 27.5°, θ_min_ = 3.4°
Absorption correction: analytical [Numeric absorption correction using a multifaceted crystal model based on expressions derived by Clark & Reid (1995) implemented in CrysAlisPro (Rigaku OD, 2015). Empirical absorption correction using spherical harmonics, implemented in SCALE3 ABSPACK]	*h* = −9→9
*T*_min_ = 0.447, *T*_max_ = 0.645	*k* = −19→20
15835 measured reflections	*l* = −15→13

### Refinement

**Table:**

Refinement on *F*^2^	Primary atom site location: dual
Least-squares matrix: full	Hydrogen site location: mixed
*R*[*F*^2^ > 2σ(*F*^2^)] = 0.016	H atoms treated by a mixture of independent and constrained refinement
*wR*(*F*^2^) = 0.038	*w* = 1/[σ^2^(*F*_o_^2^) + (0.0179*P*)^2^ + 0.7231*P*] where *P* = (*F*_o_^2^ + 2*F*_c_^2^)/3
*S* = 1.06	(Δ/σ)_max_ = 0.002
2908 reflections	Δρ_max_ = 0.97 e Å^−^^3^
194 parameters	Δρ_min_ = −0.83 e Å^−^^3^
0 restraints	

### Special details

**Table:**

Geometry. All esds (except the esd in the dihedral angle between two l.s. planes) are estimated using the full covariance matrix. The cell esds are taken into account individually in the estimation of esds in distances, angles and torsion angles; correlations between esds in cell parameters are only used when they are defined by crystal symmetry. An approximate (isotropic) treatment of cell esds is used for estimating esds involving l.s. planes.

### Fractional atomic coordinates and isotropic or equivalent isotropic displacement parameters (Å^2^)

**Table:**

	*x*	*y*	*z*	*U*_iso_*/*U*_eq_	
W1	0.45262 (2)	0.57004 (2)	0.26707 (2)	0.01171 (4)	
F1	0.4234 (2)	0.44319 (10)	0.29168 (15)	0.0209 (3)	
F2	0.4618 (2)	0.57848 (10)	0.42837 (14)	0.0226 (4)	
F3	0.7145 (2)	0.54615 (11)	0.29703 (15)	0.0222 (3)	
F4	0.4359 (2)	0.53785 (11)	0.11094 (13)	0.0233 (3)	
F5	0.1857 (2)	0.56900 (10)	0.24578 (15)	0.0202 (3)	
O1	0.4729 (3)	0.67755 (13)	0.24458 (18)	0.0242 (4)	
N1	0.1998 (3)	0.35275 (14)	0.41042 (18)	0.0134 (4)	
H1	0.286 (4)	0.3659 (19)	0.368 (2)	0.014 (7)*	
N2	0.4933 (3)	0.24312 (14)	0.39891 (19)	0.0154 (4)	
C1	0.0520 (4)	0.40604 (17)	0.4040 (2)	0.0172 (5)	
H1A	0.041718	0.455156	0.354409	0.021*	
C2	−0.0870 (4)	0.39002 (18)	0.4694 (2)	0.0198 (6)	
H2	−0.195569	0.426242	0.462155	0.024*	
C3	−0.0649 (4)	0.32076 (17)	0.5449 (2)	0.0170 (5)	
H3	−0.156152	0.310602	0.592588	0.020*	
C4	0.0917 (4)	0.26498 (17)	0.5518 (2)	0.0151 (5)	
C5	0.2231 (3)	0.28172 (16)	0.4800 (2)	0.0129 (5)	
C6	0.3802 (3)	0.22474 (16)	0.4765 (2)	0.0133 (5)	
C7	0.4049 (3)	0.15380 (17)	0.5530 (2)	0.0153 (5)	
C8	0.5628 (4)	0.10033 (18)	0.5500 (2)	0.0189 (5)	
H8	0.588937	0.052531	0.601602	0.023*	
C9	0.6790 (4)	0.11795 (18)	0.4715 (2)	0.0204 (6)	
H9	0.785780	0.082457	0.467828	0.024*	
C10	0.6365 (4)	0.18924 (17)	0.3973 (2)	0.0190 (5)	
H10	0.715470	0.199719	0.342053	0.023*	
C11	0.1198 (4)	0.19063 (17)	0.6274 (2)	0.0181 (5)	
H11	0.032761	0.178632	0.677319	0.022*	
C12	0.2702 (4)	0.13793 (18)	0.6272 (2)	0.0187 (5)	
H12	0.287124	0.089145	0.677491	0.022*	

### Atomic displacement parameters (Å^2^)

**Table:**

	*U*^11^	*U*^22^	*U*^33^	*U*^12^	*U*^13^	*U*^23^
W1	0.01223 (6)	0.01049 (6)	0.01259 (6)	−0.00060 (3)	0.00287 (4)	0.00057 (4)
F1	0.0272 (8)	0.0131 (8)	0.0256 (8)	−0.0023 (6)	0.0129 (7)	−0.0004 (6)
F2	0.0277 (9)	0.0254 (9)	0.0147 (8)	−0.0001 (7)	0.0044 (7)	−0.0030 (6)
F3	0.0153 (8)	0.0213 (8)	0.0298 (9)	0.0018 (6)	0.0037 (7)	0.0017 (7)
F4	0.0282 (9)	0.0284 (9)	0.0145 (7)	−0.0049 (7)	0.0071 (6)	−0.0014 (7)
F5	0.0121 (7)	0.0233 (9)	0.0249 (8)	0.0003 (6)	0.0027 (6)	−0.0025 (6)
O1	0.0249 (10)	0.0152 (10)	0.0320 (11)	−0.0003 (8)	0.0043 (9)	0.0021 (8)
N1	0.0137 (10)	0.0133 (10)	0.0140 (10)	−0.0023 (8)	0.0051 (9)	−0.0005 (8)
N2	0.0162 (10)	0.0131 (11)	0.0179 (11)	−0.0016 (8)	0.0058 (9)	0.0000 (9)
C1	0.0209 (13)	0.0119 (12)	0.0185 (13)	0.0018 (10)	0.0029 (11)	0.0012 (10)
C2	0.0179 (13)	0.0183 (14)	0.0238 (14)	0.0031 (11)	0.0056 (11)	−0.0047 (11)
C3	0.0167 (12)	0.0166 (12)	0.0191 (13)	−0.0019 (10)	0.0065 (10)	−0.0060 (10)
C4	0.0151 (12)	0.0154 (13)	0.0143 (12)	−0.0044 (10)	0.0017 (10)	−0.0038 (10)
C5	0.0131 (11)	0.0107 (12)	0.0142 (11)	−0.0015 (9)	0.0007 (9)	−0.0036 (10)
C6	0.0127 (11)	0.0126 (12)	0.0143 (11)	−0.0022 (9)	0.0018 (9)	−0.0027 (9)
C7	0.0158 (12)	0.0133 (12)	0.0151 (12)	−0.0025 (10)	−0.0011 (10)	−0.0013 (10)
C8	0.0210 (13)	0.0139 (12)	0.0189 (13)	0.0024 (11)	−0.0035 (11)	0.0010 (11)
C9	0.0185 (13)	0.0168 (14)	0.0250 (14)	0.0034 (10)	0.0023 (11)	−0.0042 (11)
C10	0.0152 (12)	0.0200 (14)	0.0231 (13)	−0.0010 (10)	0.0072 (11)	−0.0036 (11)
C11	0.0220 (13)	0.0174 (13)	0.0167 (12)	−0.0040 (10)	0.0083 (11)	−0.0001 (10)
C12	0.0232 (13)	0.0176 (13)	0.0143 (12)	−0.0047 (11)	0.0008 (10)	0.0018 (10)

### Geometric parameters (Å, º)

**Table:**

W1—F1	2.0048 (15)	C3—C4	1.407 (4)
W1—F2	1.8724 (16)	C4—C5	1.400 (4)
W1—F3	1.8779 (15)	C4—C11	1.441 (4)
W1—F4	1.8677 (15)	C5—C6	1.438 (4)
W1—F5	1.8809 (15)	C6—C7	1.405 (4)
W1—O1	1.698 (2)	C7—C8	1.409 (4)
N1—H1	0.89 (3)	C7—C12	1.437 (4)
N1—C1	1.334 (3)	C8—H8	0.9500
N1—C5	1.359 (3)	C8—C9	1.379 (4)
N2—C6	1.357 (3)	C9—H9	0.9500
N2—C10	1.326 (3)	C9—C10	1.400 (4)
C1—H1A	0.9500	C10—H10	0.9500
C1—C2	1.389 (4)	C11—H11	0.9500
C2—H2	0.9500	C11—C12	1.353 (4)
C2—C3	1.378 (4)	C12—H12	0.9500
C3—H3	0.9500		
			
F2—W1—F1	84.80 (7)	C3—C4—C11	122.8 (2)
F2—W1—F3	89.33 (7)	C5—C4—C3	118.3 (2)
F2—W1—F5	88.24 (7)	C5—C4—C11	118.9 (2)
F3—W1—F1	84.73 (7)	N1—C5—C4	119.2 (2)
F3—W1—F5	167.69 (7)	N1—C5—C6	119.2 (2)
F4—W1—F1	83.59 (7)	C4—C5—C6	121.6 (2)
F4—W1—F2	168.37 (7)	N2—C6—C5	117.5 (2)
F4—W1—F3	90.06 (7)	N2—C6—C7	124.7 (2)
F4—W1—F5	89.89 (7)	C7—C6—C5	117.7 (2)
F5—W1—F1	83.04 (7)	C6—C7—C8	116.6 (2)
O1—W1—F1	178.86 (8)	C6—C7—C12	120.0 (2)
O1—W1—F2	95.67 (8)	C8—C7—C12	123.5 (2)
O1—W1—F3	96.31 (8)	C7—C8—H8	120.3
O1—W1—F4	95.93 (9)	C9—C8—C7	119.5 (2)
O1—W1—F5	95.94 (8)	C9—C8—H8	120.3
C1—N1—H1	117.1 (19)	C8—C9—H9	120.7
C1—N1—C5	122.7 (2)	C8—C9—C10	118.6 (2)
C5—N1—H1	120.1 (19)	C10—C9—H9	120.7
C10—N2—C6	116.2 (2)	N2—C10—C9	124.4 (2)
N1—C1—H1A	119.9	N2—C10—H10	117.8
N1—C1—C2	120.3 (2)	C9—C10—H10	117.8
C2—C1—H1A	119.9	C4—C11—H11	120.0
C1—C2—H2	120.5	C12—C11—C4	119.9 (2)
C3—C2—C1	119.0 (3)	C12—C11—H11	120.0
C3—C2—H2	120.5	C7—C12—H12	119.1
C2—C3—H3	119.8	C11—C12—C7	121.7 (2)
C2—C3—C4	120.5 (2)	C11—C12—H12	119.1
C4—C3—H3	119.8		

### Hydrogen-bond geometry (Å, º)

**Table:**

*D*—H···*A*	*D*—H	H···*A*	*D*···*A*	*D*—H···*A*
N1—H1···F1	0.89 (3)	1.88 (3)	2.704 (3)	154 (3)
